# Supplementary material for: Radiomics Combined with Multiple Machine Learning Algorithms in Differentiating Pancreatic Ductal Adenocarcinoma from Pancreatic Neuroendocrine Tumor: More Hands Produce a Stronger Flame
Source: J Clin Med. 2022 Nov 16;11(22):6789. doi: 10.3390/jcm11226789 (PMC9697420; doi:10.3390/jcm11226789)
Supplement: Supplementary file 1 [file jcm-11-06789-s001.zip › jcm-1988738-supplementary.pdf]

The contrast agent we used was iohexol (300 mg iodine/mL; Bayer Schering Pharma AG, Leverkusen, Germany). The contrast agent was intravenously administered to patients prior to the contrast-enhanced CT scan to a total dose according to the dose-to-weight band (1.5 mL/kg). The contrast agent was injected at a constant rate of 2.5–3.0 mL/s using a dual-syringe power injector (Stellant D Dual Syringe, Medrad, Indianola, PA, USA). The scanning was performed using a single 64-detector row scanner (Brilliance 64, Philips Medical Systems, Eindhoven, Netherlands). Scanning delays used in these dual-phase protocols were 30-35 seconds for early or arterial phase and 60-70 seconds for portal venous phase. Images obtained and analyzed were from the portal venous phase. The uniform scan parameters were: beam pitch, 0.891; tube voltage, 120kVp; tube current, 200mAs; detector collimation, 0.75mm; slice thickness, 1.0mm; reconstruction increment, 5.0mm; rotation time, 0.42s; and matrix, 512x512.
